# Empowerment group therapy for refugees with affective disorders: results of a multicenter randomized controlled trial

**DOI:** 10.1192/j.eurpsy.2023.2431

**Published:** 2023-07-17

**Authors:** Maren Wiechers, Michael Strupf, Malek Bajbouj, Kerem Böge, Carine Karnouk, Stephan Goerigk, Inge Kamp-Becker, Tobias Banaschewski, Michael Rapp, Alkomiet Hasan, Peter Falkai, Andrea Jobst-Heel, Ute Habel, Thomas Stamm, Andreas Heinz, Andreas Hoell, Max Burger, Tilmann Bunse, Edgar Hoehne, Nassim Mehran, Franziska Kaiser, Eric Hahn, Paul Plener, Aline Übleis, Frank Padberg

**Affiliations:** 1 Department of Psychiatry and Psychotherapy, University Hospital LMU, Munich, Germany; 2 Department of Psychiatry and Psychotherapy, Charité - Universitätsmedizin, Campus Benjamin Franklin, Berlin, Germany; 3 Department of Psychological Methodology and Assessment, Ludwig-Maximilians-University, Munich, Germany; 4 Charlotte Fresenius Hochschule, University of Applied Sciences, Munich, Germany; 5 Department of Child and Adolescent Psychiatry, Philipps-University Marburg, Marburg, Germany; 6 Department of Child and Adolescent Psychiatry and Psychotherapy, Central Institute of Mental Health, Medical Faculty Mannheim, University of Heidelberg, Mannheim, Germany; 7 Social and Preventive Medicine, University of Potsdam, Potsdam, Germany; 8 Department of Psychiatry, Psychotherapy and Psychosomatics, Medical Faculty, University of Augsburg, BKH Augsburg, Augsburg, Germany; 9 Department of Psychiatry and Psychotherapy, RWTH Aachen University, Aachen, Germany; 10 Brandenburg Medical School, Neuruppin, Germany; 11 Department of Psychiatry and Psychotherapy, Central Institute for Mental Health, Medical Faculty Mannheim, Mannheim, Germany; 12 Department of Child and Adolescent Psychiatry and Psychotherapy, University of Ulm, Ulm, Germany

**Keywords:** Refugees, affective disorders, depression, group therapy, global mental health

## Abstract

**Background:**

Against the background of missing culturally sensitive mental health care services for refugees, we developed a group intervention *(Empowerment*) for refugees at level 3 within the stratified Stepped and Collaborative Care Model of the project *Mental Health in Refugees and Asylum Seekers* (MEHIRA). We aim to evaluate the effectiveness of the *Empowerment* group intervention with its focus on psychoeducation, stress management, and emotion regulation strategies in a culturally sensitive context for refugees with affective disorders compared to treatment-as-usual (TAU).

**Method:**

At level 3 of the MEHIRA project, 149 refugees and asylum seekers with clinically relevant depressive symptoms were randomized to the *Empowerment* group intervention or TAU. Treatment comprised 16 therapy sessions conducted over 12 weeks. Effects were measured with the Patient Health Questionnaire-9 (PHQ-9) and the Montgomery–Åsberg Depression Rating Scale (MÅDRS). Further scales included assessed emotional distress, self-efficacy, resilience, and quality of life.

**Results:**

Intention-to-treat analyses show significant cross-level interactions on both self-rated depressive symptoms (PHQ-9; *F*_(1,147)_ = 13.32, *p* < 0.001) and clinician-rated depressive symptoms (MÅDRS; *F*_(1,147)_ = 6.91, *p* = 0.01), indicating an improvement in depressive symptoms from baseline to post-intervention in the treatment group compared to the control group. The effect sizes for both scales were moderate (*d* = 0.68, 95% CI 0.21–1.15 for PHQ-9 and *d* = 0.51, 95% CI 0.04–0.99 for MÅDRS).

**Conclusion:**

In the MEHIRA project comparing an SCCM approach versus TAU, the *Empowerment* group intervention at level 3 showed effectiveness for refugees with moderately severe depressive symptoms.

## Introduction

Estimates assume that in 2023, the number of people forcibly displaced will, for the first time in history, cross the number of 117 million [1]. Studies show repeatedly higher prevalence rates of mental distress in refugee populations compared to native-borns [2, 3] and economic migrants [4], including rates for posttraumatic stress disorders and affective disorders [5]. Current group therapy approaches address different consequences of displacement-related trauma in refugees by focusing on psychoeducation [6], stabilization [7], trauma narrative and cognitive restructuring [8], or transdiagnostic processes such as impulsivity [9]. To the best of our knowledge, there is no manual targeting the treatment of depressive symptoms in refugees. We developed the *Empowerment* manual, the first depression-specific intervention for refugees [10]. The intervention comprises 16 sessions, each starting with a mindfulness or breathing exercise. Sessions 1–5 focus on psychoeducation and behavioral activation in the context of displacement. A culturally sensitive explanatory model taking pre- and post-migration stressors into account is developed [11]. Sessions 6–10 impart coping skills in dealing with migration-related acute stress, disturbed sleep, and somatic pain. Sessions 11–14 focus on emotion regulation strategies. Strategies for dealing with fear, anger, and homesickness are imparted. In the final two sessions, information about further treatment options within the German mental health care system is given. Developing the manual according to the core dimensions of cultural-sensitive psychotherapy [11] and in close cooperation with cultural mediators, we aimed to develop a manual sensitive to the cultural background and needs of refugees. The intervention was specifically developed for Arabic and Dari/Farsi-speaking refugee population groups coming from Syria, Afghanistan, Iraq, and Iran. All four countries were represented in Germany in 2014 among the 10 countries of origin with the highest inflow. Opportunities for behavioral activation and sleep hygiene in mass shelters, the inclusion of religion and cultural values (e.g., family cohesion), culturally sensitive group compositions of participants, and the use of linguistic and cultural mediators represent measures to make the intervention engaging and helpful for refugees.

The Empowerment manual was implemented for the first time within the project *Mental Health in Refugees and Asylum Seekers (MEHIRA)*, a trial developing and implementing a stratified Stepped and Collaborative Care Model (SCCM) for refugees with depressive disorders [12]. Within the SCCM, refugees received culturally sensitive interventions, with the intensity of treatment being tailored to the symptom burden. Treatment within the SCCM resulted in a more effective and cost-effective improvement in depressive symptoms compared to a treatment-as-usual group (TAU) control group [13]. Our study aimed to investigate the effectiveness of the Empowerment intervention within the framework of the MEHIRA project. The group-based therapy approach of the Empowerment intervention is presumably more cost-effective and scalable than individual therapy (level 4 of the MEHIRA SCCM), yet possibly more effective than peer-to-peer approaches (level 2 of the MEHIRA SCCM), making it an appropriate therapeutic approach to be used as part of a stepped care model. Our primary hypothesis was that the intervention is more effective in the reduction of self-rated severity of depressive symptoms compared to routine care at the time of post-intervention. Our secondary hypotheses stated that group therapy is effective in improving clinician-rated depression severity, self-efficacy, emotional distress, resilience, and quality of life in comparison to routine care.

## Methods

### Study design

Patients with moderate depressive symptoms were randomly assigned on level 3 of the SCCM to either the Empowerment intervention or TAU [12]. Randomization was carried out in a 1:1 scheme with a fixed block size using a computer-generated electronic case report form (eCRF) generated by the Clinical Study Center Berlin. All procedures contributing to this work comply with the Good Clinical Practice guidelines and with the Helsinki Declaration of 1975, as revised in 2008. All procedures involving patients were approved by the ethics committee of the Ludwig Maximilians University Munich (approval number 17-883) and the ethics boards of all other study sites. The MEHIRA project was registered in ClinicalTrials.gov (registration number: NCT03109028; registration date November 4, 2017).

### Participants

The inclusion criteria for participants of this analysis were (a) legal status of an asylum seeker or refugee [14], (b) between 18 and 65 years of age, (c) native speaking in Arabic or Dari/Farsi and/or fluent in German or English, and (d) a screening sum score between 15 and 19 on the Patient Health Questionnaire-9 (PHQ-9) [15], indicating moderate depressive symptoms. Patients were not eligible to participate in a study with (1) a current or past psychotic or degenerative disorder, (2) absent informed consent, and (3) a score of ≥4 on item 10 of the Montgomery–Åsberg Depression Rating Scale (MÅDRS) [16], indicating a current risk of suicidality. Potential participants were recruited from refugee shelters, general practitioners’ practices, and refugee educational facilities. Sample size calculation for the MEHIRA project yielded a planned sample size of 476 participants (238 per arm) for the primary outcome from baseline (*t*_0_) to time of post-intervention (*t*_1_) [12].

### Procedures

Potential participants were screened for relevant depressive symptoms and signs of emotional distress using the PHQ-9 [15] and the Refugee Health Screener (RHS-15) [17]. Participants needed to score “several days” or higher on at least five items of the PHQ-9 and attain a sum score of ≥12 on items 1–14 or a distress thermometer score of ≥5 on the RHS-15. All study-related written content was provided in German, Arabic, or Dari/Farsi. After written informed consent was obtained, symptomatology at baseline was assessed using PHQ-9 [15], RHS-15 [17], and the MÅDRS [16]. Further outcome scales included were the Brief Resilience Scale (BRS) [18], the Generalized Self-Efficacy Scale (GSE) [19], the Strength and Difficulties Questionnaire (SDQ) [20], and the World Health Organization Quality of Life Assessment (WHOQoL-BREF) [21]. Participants were then randomly assigned to receive the Empowerment intervention within the SCCM or to remain in existing routine care practices (TAU). All outcome scales were assessed at baseline (*t*_0_), at the time of post-intervention after 12 weeks (*t*_1_), at follow-up 1 after 24 weeks (*t*_2_), and at follow-up 2 after 48 weeks (*t*_3_). Data measurements were performed by independent raters blinded to the study condition while randomization, communication of group condition, and treatment were performed by unblinded study staff. To ensure blinding, the scales collected were handed over to an unblinded colleague after a rating, who then carried out the randomization in the eCRF and informed the study participants of the result of the randomization.

### Intervention

The Empowerment group intervention is a manualized group therapy written in German, designed to be carried out with the help of linguistic and cultural mediators. The manual is based on well-established cognitive behavioral therapy (CBT) principles and consists of four central components: psychoeducation, behavioral activation, stress management, and emotion regulation. The 16 Empowerment sessions were conducted over a period of 3 months. Participants attended two sessions per week in the first 4 weeks of treatment and one session per week in the last 8 weeks of treatment. The session length was 90 minutes. Group assignment was based on the same native language of the participants. In some groups, participants spoke the same language but came from different countries of origin (e.g., Arabic-speaking participants from Syria and Iraq). The translations were in Arabic or in Dari/Farsi. All groups except for one were implemented with the assistance of linguistic and cultural mediators. In this one group, the therapist herself was a native speaker of Arabic. The duration of therapy in this group was adjusted accordingly and reduced to 60 minutes per session. Groups were held with only female, only male, or mixed-gender participants. Group size was intended to be between 4 and 10 participants.

All study therapists had completed a master’s degree and were in advanced practical post-graduate training. In addition, all therapists had prior experience in therapeutic work with refugees and culturally sensitive psychotherapy. All psychologists were trained for 1 day in using the manual and working with linguistic and cultural mediators. Regular supervision sessions in-person and via phone were conducted to ensure adherence to the treatment protocol and therapy manual. Participants in the control condition received the available routine care with no stipulations made regarding the treatment received (TAU).

### Outcome measures

#### Primary outcome

The primary outcome was self-rated depression severity at post-intervention assessed by the PHQ-9. The self-rating instrument assesses depressive symptoms on a 4-point Likert scale resulting in sum scores between 0 and 27 [15]. The scale provides a test–retest reliability of 0.84 and an internal consistency of α = 0.86–0.89 [15]. Validated across different populations and cultural settings [22], the PHQ-9 is recommended by the Diagnostic and Statistical Manual of Mental Disorders, Fifth Edition (DSM-5) to be used as a general measure of depression severity.

#### Secondary outcomes

In brief, secondary outcome measures were as follows: the MÅDRS, assessing clinician-rated depression severity [16], the RHS-15 as a screening instrument for depressive symptoms, anxiety, and trauma-related disorders in refugees and asylum seekers [17], the BRS assessing the ability to recover from stress and adversity [18], the General Self-Efficacy Scale assessing patients’ sense of effective personal action control [19], the Strengths and Difficulties Questionnaire assessing emotional and behavioral problems [20], and the WHOQoL-BREF assessing patient’s quality of life [21]. Further descriptions and characteristics of these measures are reported in the Supplementary Material.

### Statistical analysis

The primary analyses were carried out on the intention-to-treat (ITT) sample, prespecified as all randomized participants for whom baseline data were available for the primary outcome. All analyses were then run with the per protocol (PP) sample, which was pre-specified as all randomized patients who attended 50% or more of the therapy sessions provided. We fitted linear mixed models (LMMs) with three hierarchical levels: time of measurement on level 1, nested within patient on level 2, and nested within study centers on level 3. The model included time (from *t*_0_ to *t*_1_) as a continuous growth factor on level 1 and condition (intervention versus TAU) as a predictor variable on level 2 to modulate cross-level interactions (time*group). We did not impute missing values in any of the analyses.

Standardized effect sizes (Cohen’s *d*) were computed for all comparisons between groups. Using logistic regression models, response and remission rates were compared across both groups for the two depression-specific outcomes PHQ-9 and MÅDRS. Response was defined as a ≥ 50% reduction of sum scores on both PHQ-9 and MÅDRS from baseline to post-intervention [23, 24]. Respectively, participants with a sum score of <5 on the PHQ-9 [25] and ≤ 10 on the MÅDRS [26] at the time of post-intervention were classified as remitters. χ^2^ tests, independent *t*-tests, or Mann–Whitney *U* tests were calculated to assess any differences between treatment groups regarding sociodemographic data and outcome scores at baseline. All tests were run using a two-sided α level of 0.05. Analyses were run with R version 4.0.5 [27].

## Results

### Patient flow

**Figure 1.**
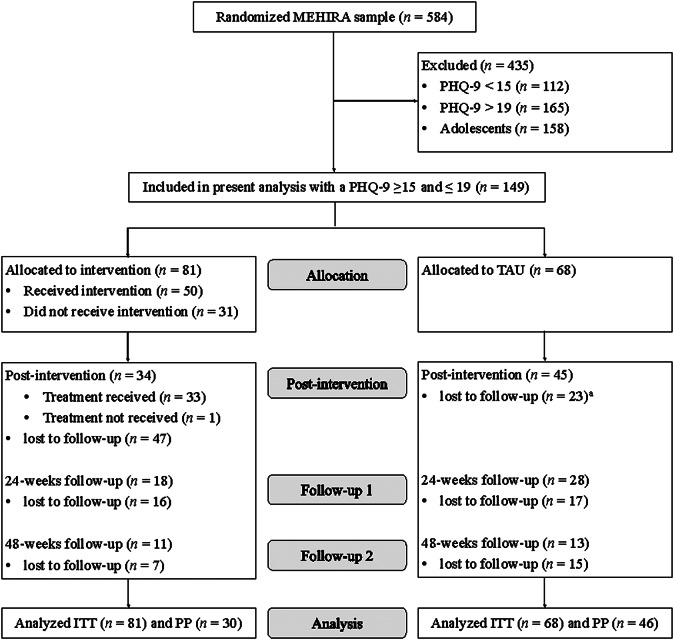
CONSORT flow chart. ITT, intention-to-treat; MEHIRA, mental health in refugees and asylum seekers; PP, per protocol; PHQ-9, patient health questionnaire-9; TAU, treatment-as-usual. ^a^No post-intervention measurements but follow-up measurements were available for one control participant.

Between April 2018 and December 2019, 584 participants were included in the MEHIRA project. The subsample for the analysis of MEHIRA level 3 (i.e., *Empowerment* versus TAU) was obtained by extracting adult participants with moderately severe depressive symptoms (PHQ-9 sum score: 15–19). In the ITT sample, 149 participants were randomly assigned to the intervention (*n* = 81) or the control group (*n* = 68). For the PP sample, only patients who had attended at least 50% of the therapy sessions were included in the analysis. Reasons why participants did not receive the Empowerment intervention or dropped out of the intervention early included having second thoughts about group therapy, deciding that they did not need therapy, having to move due to regulatory requirements or the group not taking place due to insufficient number of participants. Patient flow is presented in Figure 1.

### Drop-out analyses

Dropout rates between intervention and control groups showed significantly higher dropout rates in the intervention group at the time of post-intervention, χ^2^ (1) = 4.97, *p* = 0.026, and at the time of follow-up, χ^2^ (1) = 4.56, *p* = 0.033. Dropout rates between both groups did not differ at time of follow-up 2, χ^2^ (1) = 0.46, *p* = 0.50. No significant differences in age, sex, and baseline PHQ-9 sum score were found between dropouts and non-dropouts at time at any measurement time point (all *p* > 0.05). One reason for the high dropout rate in the intervention group was the fact that 38% of subjects had not participated in the Empowerment intervention as planned. Of those participants that had not received the intervention as indicated, all but one dropped out of the study by the time of post-intervention. Reasons why participants did not receive treatment included (1) having second thoughts about group therapy, for example, the idea that the treatment offered may not sufficiently address daily demands (e.g., poor living conditions), (2) the group not taking place due to an insufficient number of participants at the respective time point, and (3) having to move due to regulatory requirements or a rejected asylum application. Missing values were not imputed in any of the analyses.

### Baseline characteristics

Demographic and clinical data for both ITT and PP samples are presented in Table 1. The two study groups did not differ significantly from one another on any of the characteristics.

**Table 1.** Demographic and clinical characteristics upon study admission

	ITT (*n* = 149)		PP (*n* = 76)	
	Intervention	TAU	Intervention	TAU
	(*n* = 81)	(*n* = 68)	(*n* = 30)	(*n* = 46)
*Demographic characteristics*				
Age in years, mean (SD)	32.62 (9.08)	31.64 (9.84)	31.87 (8.98)	32.57 (10.80)
Female, *N*/total *N* (%)	35/81 (43.2)	22/68 (32.4)	14/30 (46.6)	13/46 (28.3)
Marital status, *N*/total *N* (%)				
Single	31/81 (38.3)	30/67 (44.8)	13/30 (43.3)	18/30 (60.0)
Married	38/81)46.9)	23/67 (34.3)	12/30 (40.0)	17/30 (56.7)
Divorced	9/81 (11.1)	10/67 (14.9)	5/30 (16.7)	8/30 (26.7)
Widowed	3/81 (3.7)	4/67 (6.0)	0/30 (0.0)	3/30 (10.0)
Having children, *N*/total *N* (%)	42/81 (51.9)	31/65 (47.7)	14/30 (46.7)	23/46 (50.0)
Education, mean (SD)	8.8 (4.4)	8.8 (4.7)	7.7 (4.2)	8.3 (4.8)
Social status change, mean (SD)	−1.2 (1.2)	−1.1 (1.2)	−0.9 (1.2)	−1.1 (1.0)
Identification migrant, mean (SD)	1.7 (1.1)	1.7 (1.2)	1.6 (1.1)	1.7 (1.1)
Religious affiliation, *n*/*n* total (%)	67/79 (84.8)	55/67 (82.1)	27/30 (90.0)	38/46 (82.6)
Residence status, *N*/total *N* (%)^a^				
Permanent residence permit	3/81 (3.7)	3/66 (4.5)	1/30 (3.3)	2/46 (4.3)
Temporary residence permit	73/81 (90.2)	54/66 (81.8)	29/30 (76.7)	40/46 (87.0)
Permanent residence in the EU	1/81 (1.2)	4/66 (6.1)	0/30 (0.0)	2/46 (33.3)
No legal residence permit	3/81 (3.7)	3/66 (4.5)	0/30 (0.0)	1/46 (2.2)
Other	1/81 (1.2)	2/66 (3.1)	0/30 (0.0)	1/46 (2.2)
Living situation, *N*/total *N* (%)				
Private flat	32/81 (39.5)	19/66 (28.8)	12/30 (40.0)	13/45 (28.9)
Refugee accommodation^b^	40/81 (49.4)	35/66 (53.0)	16/30 (53.3)	26/45 (57.8)
Shared flat	8/81 (9.9)	10/66 (15.2)	2/30 (6.7)	5/45 (11.1)
Other	1/81 (1.2)	2/66 (3.0)	0/30 (0.0)	1/45 (2.2)
Current employment				
Unemployed	70/78 (89.7)	56/66 (84.8)	27/30 (90.0)	37/46 (80.4)
Employed	8/78 (10.3)	10/66 (15.2)	3/30 (10.0)	9/46 (19.6)
Reasons for migration, *N*/total *N* (%)^c^				
War	49/81 (60.5)	44/68 (64.7)	21/30 (70.0)	30/46 (65.2)
Natural disaster	0/81 (0.0)	1/68 (1.5)	0/30 (0.0)	0/46 (0.0)
Economic crisis	6/81 (7.4)	9/68 (13.2)	4/30 (13.3)	5/46 (10.9)
Individual situation	10/81 (12.3)	12/68 (17.6)	2/30 (6.7)	8/46 (17.4)
Persecution	28/81 (34.6)	28/68 (41.2)	9/30 (30.0)	17/46 (37.0)
Social situation	18/81 (22.2)	18/68 (26.5)	7/30 (23.3)	9/46 (19.6)
Other	6/81 (7.4)	0/68 (0.0)	1/30 (3.3)	0/46 (0.0)
*Clinical characteristics*				
Subtype of depression, *n* (%)^d^				
Unipolar depression	48/79 (60.8)	35/63 (55.5)	18/30 (60.0)	22/45 (48.9)
Recurrent depressive disorder	18/79 (22.8)	20/63 (31.7)	6/30 (20.0)	17/45 (37.8)
Dysthymia	1/79 (1.3)	3/63 (4.8)	0/30 (0.0)	2/45 (4.4)
Bipolar	1/79 (1.3)	0/63 (0.0)	0/30 (0.0)	0/45 (0.0)
No diagnosis according to M.I.N.I.^e^	11/79 (13.9)	5/63 (7.9)	6/30 (20.0)	4/45 (8.9)
Reported traumatic events, mean (SD)	10.05 (6.35)	10.53 (6.35)	10.48 (6.40)	10.50 (6.12)
One comorbid axis I disorder, *n* (%)	28/79 (35.4)	16/63 (25.4)	12/30 (40.0)	11/45 (24.4)
≥ 2 comorbid axis I disorders, *n* (%)	20/79 (25.3)	20/63 (31.7)	7/30 (23.3)	15/45 (33.3)
PTSD, *n* (%)	33/79 (41.8)	22/63 (34.9)	13/30 (43.3)	18/45 (40.0)
Substance use disorder, *n* (%)	5/79 (6.3)	4/63 (6.3)	0/45 (0.0)	2/45 (4.4)
Concomitant antidepressants, *n* (%)	31/80 (38.8)	28/67 (41.8)	14 (46.7)	22/46 (47.8)
Concomitant psychotherapy, *n* (%)	15/79 (19.0)	12/66 (18.2)	5/30 (16.7)	8/45 (17.8)

Abbreviations: ITT, intention-to-treat; M.I.N.I., Mini-International-Psychiatric-Interview; *n,* number; PP, per protocol; PTSD, post-traumatic stress disorder; SD, standard deviation.

a Residence status upon study admission. Temporary residence status includes asylum seekers, asylum applicants, individuals under subsidiary protection, people under a ban on deportation and people with a tolerated right to stay. No information regarding residence status was obtained for two control participants.

b Refugee accommodation includes initial reception centers, AnkER-centers, collective accommodation centers and decentralized accommodation.

c Multiple answers possible.

d No M.I.N.I. was carried out with 7 subjects in the ITT sample and with one participant in the PP sample.

e 16 (10.7%) participants in ITT sample and 10 (13.2%) participant in the PP sample did not meet criteria for any affective disorder in the M.I.N.I.

### Primary outcome

**Figure 2.**
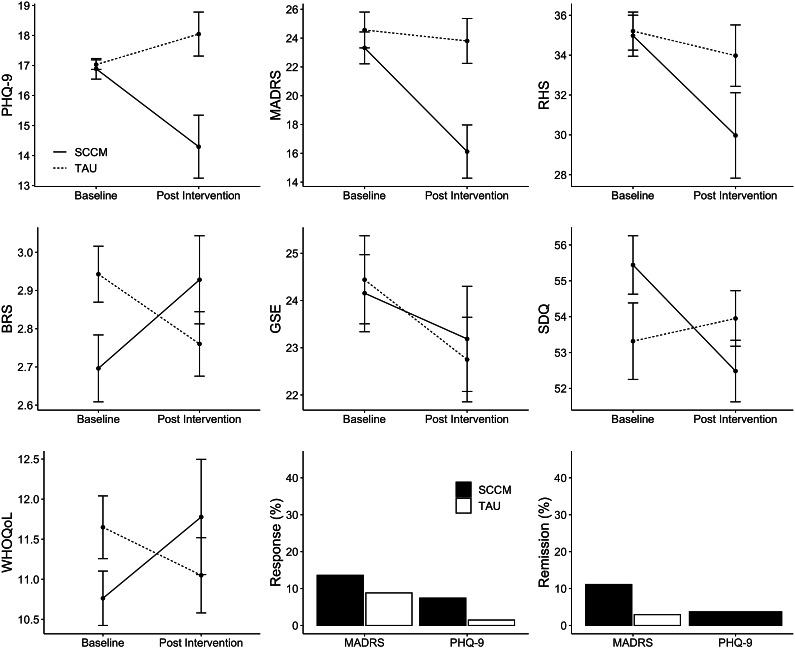
Primary and secondary outcome variables as a function of time and group within the ITT sample. BRS, brief resilience scale; GSE, general self-efficacy scale; MÅDRS, Montgomery–Åsberg depression rating scale; PHQ-9, patient health questionnaire-9; RHS, refugee health screener-15; SCCM, empowerment group intervention within the stepped and collaborative care model; SDQ, strength and difficulties questionnaire; TAU, treatment-as-usual; WHOQoL, World Health Organization quality of life questionnaire, brief version, item 1 + 2. Error bars represent ±1 standard error.

Within the ITT sample, primary outcome data were available for 149 participants at baseline (*t*_0_) and for 77 participants at post-intervention (*t*_1_). Analyses of the PHQ-9 sum scores revealed a significant time (*t*_0_ versus *t*_1_) by group (intervention versus TAU) interaction (*F*_(1,147)_ = 13.32, *p* < 0.001). Post hoc analyses revealed that Empowerment group participants showed a significant improvement in severity of depressive symptoms from baseline to post-intervention (***β*** = − 2.60, *t*_(153.62)_ = −3.59, *p* < 0.001), whereas participants in the control group showed no change in the same period (***β*** =1.03, *t*_(130.95)_ = 1.51, *p* = 0.133). Calculation of Cohen’s *d* revealed a moderate treatment effect of the intervention, *d* = 0.68 (95% CI 0.21–1.15). PHQ-9 sum score trajectories from baseline to post-intervention are presented in Table 2. Figure 2 presents PHQ-9 scores as a function of group (intervention versus TAU) and time (*t*_0_ versus *t*_1_). Results of PP analyses on the primary outcome are presented in Supplementary Table S1. Respectively for the PP sample, PHQ-9 scores as a function of group (intervention versus TAU) and time (*t*_0_ versus *t*_1_) are shown in Supplementary Figure S1.

**Table 2.** Trajectories of primary and secondary outcomes from baseline to post-intervention within ITT sample

	Intervention		TAU								
	BL	Post	BL	Post	Group		Time		Time *x* Group		ES
Outcome	*M (SD)*	*M (SD)*	*M (SD)*	*M (SD)*	*F*	*p*	*F*	*p*	*F*	*p*	*d*
PHQ-9	16.89 (3.1)	14.29 (6.11)	17.03 (1.32)	18.05 (4.81)	0.03	0.857	2.48	0.118	13.32	<0.001	0.68 (0.21 to 1.15)
MÅDRS	23.32 (9.76)	16.12 (10.61)	24.56 (9.95)	23.8 (10.45)	0.81	0.369	15.13	<0.001	6.91	0.01	0.51 (0.04 to 0.99)
RHS-15	34.98 (9.27)	29.97 (12.52)	35.21 (7.82)	33.98 (10.11)	0.02	0.901	9.04	0.003	3.39	0.068	0.44 (−0.02 to 0.9)
BRS	2.7 (0.77)	2.93 (0.65)	2.94 (0.57)	2.76 (0.53)	4.84	0.029	0.33	0.567	5	0.028	−0.42 (−0.89 to 0.06)
GSE	24.16 (7.16)	23.19 (6.3)	24.44 (7.16)	22.75 (5.66)	0.06	0.814	2.84	0.096	0.09	0.76	−0.04 (−0.51 to 0.43)
SDQ	55.44 (7.16)	52.48 (4.77)	53.32 (8.26)	53.95 (4.9)	3.11	0.08	4.61	0.035	5.68	0.02	0.58 (0.09 to 1.07)
WHOQoL-BREF (item 1 + 2)	10.76 (2.96)	11.78 (3.73)	11.65 (2.95)	11.05 (2.97)	2.64	0.106	0.12	0.726	2.71	0.103	−0.26 (−0.76 to 0.25)
WHOQoL-BREF (phys.)	44.46 (16.64)	47.98 (20.73)	43.14 (14.11)	41.13 (12.76)	1.20	0.274	0.01	0.933	0.86	0.357	−0.22 (−0.71 to 0.27)
WHOQoL-BREF (psych.)	47.74 (16.43)	40.53 (23.69)	47.54 (14.68)	38.23 (14.86)	0.01	0.928	14.34	<0.001	0.07	0.791	0.06 (−0.43to 0.56)
WHOQoL-BREF (social)	45.34 (21.63)	44.09 (27.15)	48.79 (23.17)	48.96 (20.92)	0.67	0.415	0.01	0.94	0.05	0.826	0.08 (−0.41 to 0.57)
WHOQoL-BREF (environ.)	48.75 (16.84)	52.79 (19.23)	46.44 (15.38)	49.77 (13.11)	0.70	0.403	2.67	0.106	0.00	0.954	0.08 (−0.41 to 0.56)

Abbreviations: BL, baseline; BRS, brief resilience scale; CI, confidence interval; *d*, Cohen’s *d*; ES, effect size; GSE, general self-efficacy scale; *M*, mean; MÅDRS, Montgomery–Åsberg depression rating scale; *OR*, odds ratio; PHQ-9, patient health questionnaire-9; post, post-intervention; RHS-15, refugee health screener-15; *SD*, standard deviation; SDQ, strength and difficulties questionnaire; TAU, treatment-as-usual; WHOQoL-BREF, World Health Organization quality of life questionnaire, brief version.

### Secondary outcomes

For MÅDRS as secondary outcome, the ITT sample comprised 142 participants at *t*_0_ and 78 participants at *t*_1_. Analyses reveal a main effect of time (*F*_(1,140)_ = 15.13, *p* < 0.001), as well as a time (*t*_0_ versus *t*_1_) by group (intervention versus TAU) interaction (*F*_(1,140)_ = 6.91, *p* = 0.01; Table 2). Empowerment group participants showed a significant improvement in severity of clinician-rated depressive symptoms in the same period (***β*** = − 7.27, *t*_(137.44)_ = −4.43, *p* < 0.001), whereas MÅDRS scores in the control group showed no change from baseline to post-intervention (***β*** = − 1.41, *t*_(107.28)_ = −0.934, *p* = 0.352). The intervention’s effect size was moderate (*d* = 0.51 (95% CI 0.04–0.99). At *t*_0_ and *t*_1_, data on the RHS-15 were available for 148 and 77 participants. A main effect of time indicated a reduction on RHS-15 sum scores between *t*_0_ and *t*_1_ across both groups (*F*_(1,146)_ = 9.04, *p* = 0.003). BRS scores were available at *t*_0_ for 137 participants and at *t*_1_ for 72 participants. We found a main effect of group, *F*_(1,135)_ = 4.84, *p* = 0.029, together with a time (*t*_0_ versus *t*_1_) by group (intervention versus TAU) interaction, *F*_(1,135)_ = 5, *p* = 0.028. The interaction indicated higher self-rated resilience in the group participants but not in the controls at post-intervention. Analyses of the SDQ included 137 participants at *t*_0_ and 71 participants at *t*_1_. We found a main effect of time (F_(1,135)_ = 4.61, *p* = 0.035), and a time (*t*_0_ versus *t*_1_) by group (intervention versus TAU) interaction (*F*_(1,135)_ = 5.68, *p* = 0.02). These results suggest a greater reduction in interpersonal problems in the intervention condition compared to the control group. Analyses of the WHOQoL-BREF were performed separately for the four domains physical, psychological, social, and environmental. In addition, the first two items were evaluated separately as a general indicator of quality of life. WHOQoL-BREF scores were available for 136 participants at baseline and for 71 participants at post-intervention. For the psychological domain, a main effect of time indicated a decline in psychological life quality from baseline to time of post-intervention in both groups (*F*_(1,134)_ = 14.34, *p* < 0.001). Analyses of the other domains yielded no results. Analyses of the GSE showed no significant effects. Sum score trajectories of all secondary outcome scales from baseline to post-intervention are presented in Table 2. Figure 2 presents secondary outcomes as a function of group (intervention versus TAU) and time (*t*_0_ versus *t*_1_). Results of PP analyses on the secondary outcomes are presented in supplementary Table S1. Respectively, secondary outcome scores for both groups (intervention versus TAU) and measurement times (*t*_0_ versus *t*_1_) and time are presented in Supplementary Figure S1.

Response and remission rates at *t*_1_ for PHQ-9 and MÅDRS are shown in Table 2. The response rates in the treatment group were significantly higher compared to the control group based on PHQ-9 sum scores (OR = 9, 95% CI 1.43–174.78, *p* = 0.047) and MÅDRS sum scores (OR = 3.74, 95% CI 1.15–13.62, *p* = 0.032). Group participation leads to significantly higher remission rates compared to the control group based on MÅDRS sum scores (OR = 13.55, 95% CI 2.51–118.77, *p* = 0.006).

## Discussion

We examined the effectiveness of a cultural-sensitive group intervention for refugees and asylum seekers with moderate depressive symptoms within the multicenter MEHIRA project that compares an SCCM approach versus TAU [12]. Our findings point toward the effectiveness of the intervention compared to treatment-as-usual. Participating in the group intervention resulted in a greater decrease in self-assessed and clinician-rated depressive symptomatology compared to TAU. The within-intervention effect size for both scales was moderate. Group participation resulted in significantly higher response and remission rates compared to the control group. The results are comparable to the mean effect sizes of a peer-provided problem management group intervention (PM+) for refugees with depressive and stress-related symptoms [28]. The preventive self-help group intervention SH+ developed by the WHO found small positive effects on the development of current mental disorders 2 weeks, but not 6 months, after the end of the intervention [29]. A meta-analysis evaluating the effectiveness of different interventions, including NET, EMDR, and culturally adapted CBT found medium to high effect sizes for PTSD symptoms and high effect sizes for depressive symptoms [30]. Compared to our results, a cognitive-behavioral therapy plus problem-solving (CA-CBT+) intervention for refugees greatly improved participants’ overall psychological distress. The results raise the question of whether refugee populations in particular benefit from problem-solving skills training [31].

In our study, group participants reported fewer difficulties in interpersonal relationships (SDQ) after the end of therapy, suggesting group participation to promote prosocial behavior and social skills. It may also be the group context itself that is particularly well suited for refugee patients, the majority of which have had experiences with dictatorial systems, betrayal, or torture. Throughout the course of the intervention, trusting relationships a sense of belonging, and strong cohesion in the groups often developed. Participating in the Empowerment group therapy increased patients’ resilience compared to the control group. Group participation had no effect on the participant’s quality of life. A possible explanation could be that the WHOQoL-BREF assesses areas of life that remain unaffected by the intervention but have a major impact on the life quality of people who have fled their homes (e.g., monetary needs, living conditions).

### Strengths and limitations

A key strength of our study is to include a large sample of refugees from four study sites within a randomized controlled design. Another strength is the culturally sensitive treatment approach, that specifically takes the needs and values of refugee populations into account.

We would like to address the following limitations of our study. First, data at the time of post-intervention was only available for 53% of the participants. Refugee populations often represent a very mobile group, leading to high dropout rates in clinical studies [32], and could therefore benefit from interventions that are shorter or flexible in duration. The Empowerment intervention with its 16 sessions could possibly be too long in its duration for the constantly changing circumstances of refugees, which favor drop-out rates. Second, our group intervention trial was conducted at university hospitals, a setting that is not representative of primary care in mental health. In the future, however, the intervention would be scalable for various other settings, for example, delivered by trained health care workers in low-and-middle-income counties (LAMICS) or provided as part of video-based services for outreach to rural areas. Such an Empowerment video-based group intervention has already been developed by our research team as part of a pilot study.

## Conclusion

Our study demonstrated the effectiveness of the Empowerment group intervention (i.e., level 3 of the MEHIRA SCCM) as a new treatment approach for refugees and asylum-seekers with depressive symptoms. The next step is ensuring that the intervention reaches populations in LAMICS, where resources are limited and the demand for mental health interventions is high. This implies networking with social and community health services in the respective populations and may require an adaptation of the intervention’s duration, to address the often highly mobile living circumstances of refugees. A short version of the Empowerment intervention has lately been developed for Ukraine refugees, an adaptation that could also be helpful for refugee populations in LAMICS.

## Supporting information

Wiechers et al. supplementary material Wiechers et al. supplementary material

Wiechers et al. supplementary material Wiechers et al. supplementary material

## Data Availability

The trial data can be requested deidentified and anonymized by researchers for future usage in independent scientific research projects. These requests should be addressed to the corresponding author to negotiate a data-sharing agreement with the Ludwig Maximilians University Munich.

## Supplementary material

The supplementary material for this article can be found at https://doi.org/10.1192/j.eurpsy.2023.2431.

## References

[1] Global Appeal 2023 [internet]. 2023. [cited 2023 January 5th]. Available from: https://reporting.unhcr.org/globalappeal.

[2] Nickerson A. Pathways to recovery: Psychological mechanisms underlying refugee mental health. In: Morina N, Nickerson A, editors, Mental health of refugee and conflict-affected populations. Cham: Springer; 2018, p. 91–109. doi:10.1007/978-3-319-97046-2_5

[3] Bogic M, Njoku A, Priebe S. Long-term mental health of war-refugees: a systematic literature review. BMC Int Health Hum Rights. 2015;15:1–41. doi:10.1186/s12914-015-0064-9 26510473 PMC4624599

[4] Lindert J, von Ehrenstein O, Priebe S, Mielck A, Brähler E. Depression and anxiety in labor migrants and refugees - a systematic review and meta-analysis. Soc Sci Med. 2009;69:246–57. doi:10.1016/j.socscimed.2009.04.032 19539414

[5] Hoell A, Kourmpeli E, Salize HJ, Heinz A, Padberg F, Habel U, et al. Prevalence of depressive symptoms and symptoms of post-traumatic stress disorder among newly arrived refugees and asylum seekers in Germany: Systematic review and meta-analysis. BJPsych Open. 2021;7:e93. doi:10.1192/bjo.2021.54 33938425 PMC8142547

[6] Liedl A, Schäfer U, Knaevelsrud C. Psychoedukation bei posttraumatischen Störungen: Manual für Einzel-und Gruppensetting. Stuttgart: Klett-Cotta; 2018.

[7] Özkan I, Belz M. Sprachreduzierte Ressourcen-und Traumastabilisierungsgruppe. Stuttgart: Klett-Cotta; 2019.

[8] Drožđek B, Bolwerk N. Evaluation of group therapy with traumatized asylum seekers and refugees—the Den Bosch model. Traumatology. 2010;16(4):117–27. doi:10.1177/1534765610388298

[9] Koch T, Ehring T, Liedl A. Skills-training der Affektregulation – Ein kultursensibler ansatz: STARK. Psychotherapeut. 2017;4:316–23.

[10] Wiechers M, Übleis A, Padberg F. Empowerment für menschen mit affektiven Erkrankungen und Fluchterfahrungen. Stuttgart: Schattauer; 2019.

[11] Hinton D, Patel A. Culturally sensitive CBT for refugees: key dimensions. In: Morina N, Nickerson A, editors. Mental health of refugee and conflict-affected populations. Cham: Springer; 2018, p. 201–19. doi:10.1007/978-3-319-97046-2_10

[12] Böge K, Karnouk C, Hahn E, Schneider F, Habel U, Banaschewski T, et al. Mental health in refugees and asylum seekers (MEHIRA): study design and methodology of a prospective multicentre randomized controlled trail investigating the effects of a stepped and collaborative care model. Eur Arch Psychiatry Clin Neurosci. 2020;270:95–106. doi:10.1007/s00406-019-00991-5 30796528

[13] Böge K, Karnouk C, Hoell A, Kamp-Becker I, Padberg F, Übleis A, et al. Effectiveness and cost-effectiveness for the treatment of depressive symptoms in refugees and asylum seekers: a multi-centred randomized controlled trial. Lancet Reg Health Eur. 2022;19:100413. doi:10.1016/j.lanepe.2022.100413 35694653 PMC9184853

[14] What is a refugee? [internet] [cited 2022 May 23rd, https://www.unhcr.org/what-is-a-refugee.html; 2021 [accessed 23rd May 2022].

[15] Kroenke K, Spitzer RL, Williams JBW. The PHQ-9: validity of a brief depression severity measure. J Gen Intern Med. 2001;16:606–13. doi:10.1046/j.1525-1497.2001.016009606.x 11556941 PMC1495268

[16] Montgomery S, Åsberg M. A new depression scale designed to be sensitive to change. Br J Psychiatry. 1979;134:382–9. doi:10.1192/bjp.134.4.382 444788

[17] Hollifield M, Farmer B. Effective screening for emotional distress in refugees. J Nerv Ment Dis. 2016;204:247–53. doi:10.1097/NMD.0000000000000469 26825376

[18] Smith BW, Dalen J, Wiggins K, Tooley E, Christopher P, Bernard J. The brief resilience scale: assessing the ability to bounce back. Int J Behav Med. 2008;15:194–200. doi:10.1080/10705500802222972 18696313

[19] Schwarzer R, Jerusalem M. The general self-efficacy scale (GSE). Anxiety Stress Coping 2010;12:329–45, http://userpage.fu-berlin.de/~health/selfscal.htm [accessed 15 January 2022].

[20] Goodman R. Psychometric properties of the strengths and difficulties questionnaire. J Am Ac Child Adolesc Psychiatry. 2001;40:1337–45. doi:10.1097/00004583-200111000-00015 11699809

[21] Skevington SM, Lotfy M, O’Connell KA. The World Health Organization’s WHOQOL-BREF quality of life assessment: psychometric properties and results of the international field trial. Qual Life Res. 2004;13:299–310. doi:10.1023/B:QURE.0000018486.91360.00 15085902

[22] Grupp F, Piskernik B, Mewes R. Is depression comparable between asylum seekers and native Germans? An investigation of measurement invariance of the PHQ-9. J Aff Dis. 2020;262:451–8. doi:10.1016/j.jad.2019.11.055 31744740

[23] Riedel M, Möller H J, Obermeier M, Schennach-Wolff R, Bauer M, Adli M, et al. Response and remission criteria in major depression – a validation of current practice. J Psychiatr Res. 2010;44:1063–8. doi:10.1016/j.jpsychires.2010.03.006 20447651

[24] van Diermen L, van den Ameele S, Kamperman AM, Sabbe BCG, Vermeulen T, Schrijvers D, et al. Prediction of electroconvulsive therapy response and remission in major depression: meta-analysis. Br J Psychiatry. 2018;212:71–80. doi:10.1192/bjp.2017.28 29436330

[25] McMillan D, Gilbody S, Richards D. Defining successful treatment outcome in depression using the PHQ-9: a comparison of methods. J Aff Dis. 2010;127:122–9. doi:10.1016/j.jad.2010.04.030 20569992

[26] Keller MB. Past, present, and future directions for defining optimal treatment outcome in depression: remission and beyond. JAMA. 2003;289:3152–60. doi:10.1001/jama.289.23.3152 12813121

[27] R Development Core Team. R: A language and environment for statistical computing [Software]. Vienna, Austria: R Foundation for Statistical Computing; 2011.

[28] de Graaff AM, Cuijpers P, McDaid D, Park A, Woodward A, Bryant RA, et al. Peer-provided problem management plus (PM+) for adult Syrian refugees: a pilot randomised controlled trial on effectiveness and cost-effectiveness. Epidemiol Psychiatr Sci. 2020;29:e162. 10.1017/S2045796024000453 PMC11464952 39344836

[29] Purgato M, Carswell K, Tedeschi F, Acarturk C, Anttila M, Au T, et al. Effectiveness of self-help plus in preventing mental disorders in refugees and asylum seekers in Western Europe: a multinational randomized controlled trial. Psychother Psychosom. 2021;90(6):403–14. 34350902 10.1159/000517504 PMC8619740

[30] Kip A, Priebe S, Holling H, Morina N. Psychological interventions for posttraumatic stress disorder and depression in refugees: a meta‐analysis of randomized controlled trials. Clin Psychol Psychother. 2020;27(4):489–503. 32191370 10.1002/cpp.2446

[31] Kananian S, Soltani Y, Hinton D, Stangier U. Culturally adapted cognitive behavioral therapy plus problem management (CA-CBT+) with afghan refugees: a randomized controlled pilot study. J Trauma Stress. 2020;33:928–38. doi:10.1002/jts.22615 33155348

[32] Semmlinger V, Ehring T. Preventing dropout in research, assessment and treatment with refugees - a guideline. Clin Psychol Psychother. 2022;29:767–82. doi: 10.31234/osf.io/nukv3 34585469

