# Supplementary material for: Empowerment group therapy for refugees with affective disorders: results of a multicenter randomized controlled trial
Source: Eur Psychiatry. 2023 Jul 17;66(1):e64. doi: 10.1192/j.eurpsy.2023.2431 (PMC10594347; doi:10.1192/j.eurpsy.2023.2431)
Supplement: Wiechers et al. supplementary material [file S0924933823024318sup001.docx]

**Supplement**

***Descriptions and characteristics of secondary outcomes***

Rater-based clinical interview MÅDRS measuring depression severity was included as a secondary outcome. The scale’s 10 items are measured on a 6-point likert scale, including symptoms identified as selective and sensitive to treatment response [16]. The RHS-15 is an instrument used for screening anxiety, depressive and trauma-related disorders in refugee populations [17]. 14-items assess symptoms on a scale from 0 (not at all) to 4 (extremely), while item 15 presents a distress thermometer, on which participants rank the overall distress they experienced within the last week on a scale from 0 (no distress) to 10 (extreme distress). The BRS is a six-item instrument measuring resilience [18], i.e. the ability to recover from stress and adversity. Responses are rated on a five-point likert scale ranging from 1 (very rarely or never) to 5 (very often or always). Patients sense of effective personal action control, i.e. perceived self-efficacy was assessed by the GSE [19]. Participants rate ten statements regarding their ability to cope with everyday challenges on a 4-point likert scale ranging from 1 (not at all true) to 4 (exactly true). The SDQ as a brief five factor instrument assessing emotional and behavioral problems was included to assess interpersonal difficulties in participants on a 3-point likert type scale (0=not true, 1=somewhat true, 2=certainly true). The 25-item scale comprises the five subscales emotional problems, conduct problems, hyperactivity-inattention, peer problems and prosocial behavior [20]. Life quality was measured by the WHOQoL-BREF, a 26-item version of the WHOQoL-100 assessment [21]. The scale assesses life quality across the four domains physical, psychological, social and environment on 5-point likert scales.

**Table S1.** Trajectories of primary and secondary outcomes from baseline to post-intervention within PP sample.

|  | Intervention | | | | | TAU | | | |  | | | | | | | | | | | |
| --- | --- | --- | --- | --- | --- | --- | --- | --- | --- | --- | --- | --- | --- | --- | --- | --- | --- | --- | --- | --- | --- |
|  | BL | Post | | |  |  | BL | | Post | Group | | | Time | | | | | | Time x Group | | ES |
| Outcome | *M (SD)* | *M (SD)* | | | | *M (SD)* | | | *M (SD)* | *F* | | *p* | | *F* | | | *p* | *F* | | *p* | *d* |
| *Primary outcome* | | |  |  | | | |  | |  |  | | | |  |  | | |  |  |  |
| PHQ-9 | 16.93  (3.24) | 14.31  (6.6) | | | | 16.98  (1.36) | | | 18.05  (4.81) | 0.01 | | .921 | | 1.35 | | | .249 | 8.25 | | .005 | 0.67 (0.18  to 1.16) |
| *Secondary outcomes* | | |  |  | | | |  | |  | |  | |  | | |  |  | |  |  |
| MÅDRS | 23.53  (9.26) | 16.93  (10.78) | | | | 26.25  (9.65) | | | 23.8  (10.45) | 0.76 | | .386 | | 12.06 | | | .001 | 4.06 | | .048 | 0.52 (0.03  to 1.01) |
| RHS-15 | 36.13  (9) | 28.83  (12.98) | | | | 35.7  (7.37) | | | 33.98  (10.11) | 0.25 | | .618 | | 11 | | | .001 | 4.56 | | .036 | 0.5 (0.01  to 0.98) |
| BRS | 2.69  (0.83) | 2.91  (0.69) | | | | 2.82  (0.55) | | | 2.76  (0.53) | 0.69 | | .408 | | 0.72 | | | .399 | 2.12 | | .15 | -0.37 (-0.87  to 0.13) |
| GSE | 23.66  (7.01) | 22.93  (6.62) | | | | 24  (6.89) | | | 22.75  (5.66) | 0.05 | | .816 | | 1.7 | | | .196 | 0.07 | | .798 | -0.05 (-0.55  to 0.45) |
| SDQ | 57.28  (6.19) | 52.7  (4.94) | | | | 53.98  (7.36) | | | 53.95  (4.9) | 5.09 | | .026 | | 7.53 | | | .008 | 6.83 | | .011 | 0.6 (0.09  to 1.1) |
| WHOQoL-BREF (item 1+2) | 10.9  (2.76) | 12  (3.87) | | | | 11.75  (2.53) | | | 11.09  (2.97) | 1.66 | | .2 | | 0.1 | | | .751 | 2.98 | | .089 | -0.31 (-0.8  to 0.21) |
| WHOQoL-BREF (physical) | 45.75  (14.79) | 49.18  (21.77) | | | | 42.36  (13.96) | | | 41.13  (12.76) | 0.53 | | .469 | | 0.17 | | | .683 | 1.42 | | .237 | -0.3 (-0.8  to 0.21) |
| WHOQoL-BREF (psych.) | 49.43  (15.93) | 40.99  (23.94) | | | | 46.6  (15.67) | | | 38.23  (14.86) | 0.34 | | .56 | | 11.25 | | | .001 | 0 | | .959 | 0.04 (-0.47  to 0.55) |
| WHOQoL-BREF (social) | 41.95  (20.83) | 42.59  (25.25) | | | | 46.6  (15.67) | | | 38.23  (14.86) | 0.83 | | .364 | | 0.14 | | | .714 | 0.04 | | .84 | 0.06 (-0.45  to 0.56) |
| WHOQoL-BREF (environ.) | 48.46  (15.87) | 52.78  (19.3) | | | | 46.69  (14.45) | | | 49.77  (13.11) | 0.11 | | .737 | | 2.43 | | | .124 | 0.03 | | .874 | 0.02 (-0.48  to 0.52) |

*Note.* TAU = treatment-as-usual; BL = baseline, Post = post-intervention; *M* = mean; *SD* = standard deviation; ES = effect size; *d* = Cohen’s *d*; CI = confidence interval; PHQ-9 = Patient Health Questionnaire-9; MÅDRS = Montgomery Åsberg Depression Rating Scale; RHS-15 = Refugee Health Screener-15; BRS = Brief Resilience Scale; GSE = General Self-Efficacy Scale; SDQ = Strength and Difficulties Questionnaire; WHOQoL-BREF = World Health Organization Quality of Life questionnaire, brief version.

**Figure S1.** Primary and secondary outcome variables at primary study endpoint within the PP sample.

*Note.* TAU = treatment-as-usual; SCCM = Empowerment group intervention within the Stepped and Collaborative Care Model; PHQ-9 = Patient Health Questionnaire-9; MÅDRS = Montgomery Åsberg Depression Rating Scale; RHS = Refugee Health Screener-15; BRS = Brief Resilience Scale; GSE = General Self-Efficacy Scale; SDQ = Strength and Difficulties Questionnaire; WHOQoL = World Health Organization Quality of Life questionnaire, brief version, item 1 + 2. Error bars represent ± 1 standard error.
